# Optimizing muscle power after stroke: a cross-sectional study

**DOI:** 10.1186/1743-0003-9-67

**Published:** 2012-09-27

**Authors:** Verna A Stavric, Peter J McNair

**Affiliations:** 1 School of Rehabilitation and Occupation Studies, AUT University, Private Bag 92006, Auckland, 1142, New Zealand

**Keywords:** Hemiplegia, Hemi paresis, Cerebrovascular accident, Stroke, Leg extensor, Power, Strength, Power, Rehabilitation

## Abstract

**Background:**

Stroke remains a leading cause of disability worldwide and results in muscle performance deficits and limitations in activity performance. Rehabilitation aims to address muscle dysfunction in an effort to improve activity and participation. While muscle strength has an impact on activity performance, muscle power has recently been acknowledged as contributing significantly to activity performance in this population. Therefore, rehabilitation efforts should include training of muscle power. However, little is known about what training parameters, or load, optimize muscle power performance in people with stroke. The purpose of this study was to investigate lower limb muscle power performance at differing loads in people with and without stroke.

**Methods:**

A cross-sectional study design investigated muscle power performance in 58 hemiplegic and age matched control participants. Lower limb muscle power was measured using a modified leg press machine at 30, 50 and 70% of one repetition maximum (1-RM) strength.

**Results:**

There were significant differences in peak power between involved and uninvolved limbs of stroke participants and between uninvolved and control limbs. Peak power was greatest when pushing against a load of 30% of 1RM for involved, uninvolved and control limbs. Involved limb peak power irrespective of load (Mean:220 ± SD:134 W) was significantly lower (p < 0.05) than the uninvolved limb (Mean:466 ± SD:220 W). Both the involved and uninvolved limbs generated significantly lower peak power (p < 0.05) than the control limb (Mean:708 ± SD:289 W).

**Conclusions:**

Significant power deficits were seen in both the involved and uninvolved limbs after stroke. Maximal muscle power was produced when pushing against lighter loads. Further intervention studies are needed to determine whether training of both limbs at lighter loads (and higher velocities) are preferable to improve both power and activity performance after stroke.

## Background

Stroke has a significant impact on death and disability worldwide [1-3]. People who have sustained a stroke often present with a decrease in function during activities such as walking or using stairs which, in part, which can largely be attributed to muscle performance deficits [1,2,4-8]. Such decreases in function have been associated with reduced participation in work and leisure activities and a decreased quality of life [7,9]. Muscle performance encompasses concepts of strength and power. Strength is practically assessed as the maximum load that might be lifted or the maximum torque that might be generated during a joint movement. It can be assessed during different types of muscle action (isometric, concentric and eccentric). Power is defined as the product of force and velocity and its measurement in exercise environments involves the measurement of strength and the velocity of joint movement.

There is some evidence to suggest that power may have a larger influence than strength on one’s ability to effectively undertake daily activities. Schultz [10] and Cuoco et al. [11] have proposed that, in the older adult, the performance of many activities of daily living (ADL) does not require large amounts of strength and may rely more heavily on the ability to develop the required strength quickly, that is, with greater power. Indeed, many authors investigating function in older adults have reported a significant relationship between muscle power and function. For instance, leg power was the strongest predictor of ambulatory status as compared to other variables, including strength, in a number of investigations [12-16]. Bean et al. [17] further concluded that not only does muscle power consistently explain more of the variance (22%-38%) than does strength in many activities, but also that low power would result in a two to threefold greater risk of mobility limitations than low strength. A further indication of the role muscle power plays in function can be seen in clinical trials [18,19] that have compared strength training to power training and showed that those who power trained showed greater gains in function. Furthermore, studies investigating muscle power in the stroke population have found significant relationships between lower limb power and ambulatory performance [20,21].

If power training is to be implemented in stroke rehabilitation, an understanding of the degree of power loss associated with having a stroke is needed. For the clinician in most rehabilitation centers, manipulating the load on a weights training machine as a percentage of maximal strength and instructing the patient to move his or her joints as fast as possible during the assessment is the most practical means of not only examining power deficits but also instigating training programs that can be reassessed easily. Identifying whether a person has a greater power deficit at a particular percentage of maximum strength allows the clinician to prioritize the initial exercise training program to that which would be most beneficial to improving the performance of meaningful tasks.

Loads that best elicit maximum power have been investigated in other populations. Kaneko, Fuchimoto, Toju & Suei [22] noted that maximal power in the upper limb in young healthy males occurred after testing and training at 30% of maximum isometric strength. In contrast, in the same population, Siegel, Gilders, Staron & Hagerman [23] demonstrated that, when performing a bilateral squat, maximal muscle power was produced at loads of 50-70% of maximal force. Similarly, in older adults, Fielding et al. [24] found that loads of 70% of maximum isoinertial strength generated maximum lower limb power production. These study findings, however, are in contrast to de Vos, Singh, Ross, Stavrinos, Orr & Fiatarone Singh [25] who found that older adults’ ability to generate power increased by similar amounts when training across low (20% of one repetition maximum or 1-RM), medium (50% of 1-RM) and high (80% of 1-RM) loads. Thus, to date, there is no consensus on a loading level that optimizes power performance in healthy populations.

With increasing attention and support for power training in stroke rehabilitation [20,21], clear training parameters need to be established. To date, there have been few investigations that address where, in the loading spectrum, power generation is affected most in individuals with stroke. Defining this level is the first step in identifying the initial loads that might be utilized in an exercise training program which would be particularly useful if the identified loads have relevance to meaningful aspects of activity and participation. As such, this information is needed to maximize improvements in physical function and activity performance. Hence, the primary aim of this study was to compare muscle power generated at different loads (30%, 50%, 70% of 1-RM) during an explosive leg press power task using the involved and uninvolved lower limbs of individuals with stroke and to compare those values with those generated by age and gender matched participants without stroke. A leg press/extension activity was chosen as it is a multi joint action that requires coordination and therefore is related to many activities that lower limbs perform during tasks of daily living.

## Methods

### Participants

Community dwelling adults with and without stroke were invited to participate on a voluntary basis. They were recruited from local stroke clubs, rehabilitation clinics, notices in the local newspapers and from existing participant databases. Those with stroke had a diagnosis of unilateral stroke of at least 6 months and had residual motor deficits as a result of the stroke affecting at least the lower limb. All participants were required to be able to ambulate with or without an assistive device for at least ten meters; able to communicate, with an ability to understand and follow simple commands as determined by a Mini Mental State Examination [26] score of 21 or above; and able to participate as per their responses to the Physical Activity Readiness questionnaire (PARQ) [27]. If the participant responded that they had a medical problem or were taking medications that might be detrimental to their health if they participated, they were referred to their physician for clearance. Participants were excluded if they demonstrated an inability to get onto or lift the minimal weight (4 kg) on the modified leg press machine; and, in the stroke group, if they demonstrated a score of 3 or more on the Modified Ashworth Spasticity scale of the quadriceps or plantar-flexor muscles on the affected limb. A participant informed consent form was signed prior to commencement of testing. Ethical approval was obtained from the ethics committee of the university.

Based on an effect size of 0.5, a power of 0.8, with an alpha level of 0.05, a pilot study was conducted using 10 participants to calculate sample size. The primary variable was power generated during a leg press exercise. The sample size required was 60.

### Procedure

Data was collected over two sessions separated by no less than two and no more than seven days. The first session was used for screening and descriptive and demographic data collection. This session was also used to determine each participant’s maximum strength (1-RM) for each leg. The second session was used for assessing muscle power performance at differing percentages of the previously determined maximum strength.

### Measurements of strength and power

#### Instrumentation

**Figure 1:**
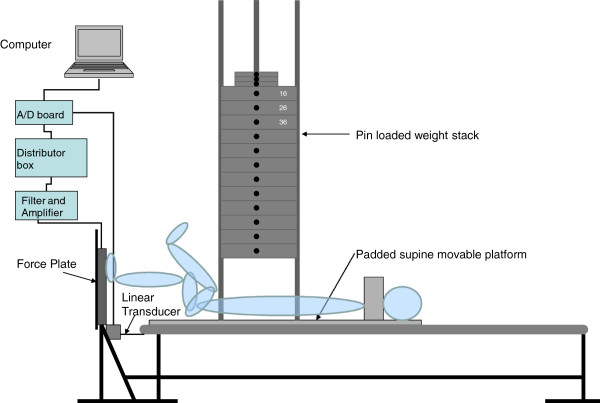
** Schematic representing the modified leg press machine and the position of the participants at the start of testing.** The tested limb is at 90 degrees of hip and knee flexion.

All muscle force and power testing occurred on a modified supine leg press machine. Details of this machine have been reported previously by Cronin, McNair and Marshall [28]. See Figure 1 for a schematic representation. 

A force plate (Advanced Mechanical Technology Inc., 179 Waltham Street, Watertown, MA 02172, USA) was mounted onto the foot plate of the modified leg press machine, perpendicular to the moving platform (see Figure 1). Force plate signals, orthogonal to the movement, were sampled at 1000 Hz, amplified and filtered with a 10.5 Hz low pass cut-off filter and relayed to a custom made data acquisition and analysis program (Super Scope II, Version 3.0, GW Instruments, Boston, USA).

A linear transducer (P-80A, Unimeasure, Oregon) was attached to the platform of the modified leg press machine. The transducer data, accurate to 0.1cm, was sampled at 1000 Hz by the above mentioned computer-based data acquisition and analysis program to provide displacement/time data. This system has been shown to be both valid and reliable in previous testing [29].

#### Leg press strength assessment

Using the modified leg press machine, unilateral lower limb maximum leg extension strength was assessed using a one-repetition maximum procedure. 1-RM refers to the maximum weight that a person can lift once through a prescribed range of motion. Specifically, this involved a 5 minute warm up on a stationary Monark bicycle pedaling at approximately 60 RPM at a 5 watt loading. Exer-cycling was chosen as it involved low level activation of the muscles of the lower limb that were involved in the leg press machine protocol. After cycling, the participants were positioned on the supine leg press machine in a standardized position with the knee and hip angle set at approximately 90° (see Figure 1). The participant’s untested lower limb was held secure via a supporting strap. From the starting position, participants were asked to extend their leg “as hard as possible”, keeping the foot in contact with the force plate, until full available knee and hip extension was reached, and then return to the starting position. In the current study, the 1-RM testing procedure was based on a protocol described by Baechle, Earle and Wathen [30]. After a set of 10 repetitions at a submaximal loading level, a clinician experienced in strength assessment selected a load that the subject could lift unilaterally for 2 to 5 repetitions. Following a 2–3 minute rest period, the load was increased by 5-15% and another 1-RM was attempted. If the subject completed one repetition they stopped and rested for at least two minutes. Then, depending on the observed effort and the participant’s perceived level of exertion, the load was increased by a further 5-15% and the 1-RM test was repeated. This process continued until the maximum load that the subject could lift, with proper technique and for only one repetition, was reached. Subjects performed 2–3 attempts to reach their 1-RM. If at any point the subject was unable to complete one full repetition, the load was decreased by 5-10% until a successful lift was completed.

#### Leg press muscle power assessment

In the second session, participants returned for power testing of each lower limb. After an identical warm up to that in session 1, participants were positioned in the leg press machine as previously described and pushed to extend each leg against 30%, 50% and 70% of the 1-RM determined in the previous session. For the power assessment, they were asked to perform the movement “as hard and as fast as possible”. After each trial, participants rested for 1 minute. Two repetitions at each load were performed. A rest of up to 3 minutes was provided when the loads were changed. Testing order of the three different loads was randomized prior to testing.

#### Muscle power calculation

Force and displacement signals were used to calculate power. The rate of change of displacement derived from the linear displacement transducer was used to calculate the velocity at which the platform moved, and this was undertaken using a differentiation algorithm. Power was subsequently calculated as the product of force and velocity. The highest power observed across the two trials at each load was used in the statistical analysis. Reliability was established in pilot testing where a small cohort of subjects (N = 8) undertook testing on 2 separate days not more than 7 days apart. The variable of interest was peak power and the ICCs generated from the data were 0.91-0.97 across loads of 30%, 50%, 70% 1-RM.

### Statistical analysis

Statistical analyses were undertaken using the Statistical Package for Social Sciences (SPSS) Version 11.0 (SPSS Inc., Illinois, USA). Descriptive data were assessed to determine the appropriateness of parametric analysis. Control and stroke groups were compared for baseline characteristics of height, weight and body mass index (BMI) using an independent t–test or, if required, a Mann–Whitney *U* test. A 3x3 analysis of variance (ANOVA) was used. The factors were load (30%, 50%, 70% of 1-RM) and limbs (stroke affected, unaffected, and the dominant control group leg). Departures from sphericity in the repeated measures were accounted for with the Huynh-Fedlt epsilon value. The Bonferroni test was utilized to assess differences across legs at each of the different loading levels [31]. An alpha level of 0.05 was set.

## Results

Ninety eight people expressed interest in the study. Of these, 58 met the inclusion criteria. Baseline characteristics of the two groups of participants, including age, height, mass and BMI are presented in Table 1. Independent t-tests examining differences between the two groups across the baseline measures showed no significant differences (p > 0.05). The male: female ratio was 17:13 for both groups

**Table 1:** Descriptive statistics (M ± SD) for age, height, mass and calculated BMI* for stroke group (n = 29) and control group (n = 29)

	**Stroke M ± SD**	**Range**	**Control M ± SD**	**Range**
**Age** (yrs)	64.6 ± 12.3	40.0 - 85.0	65.3 ± 12.2	38.0 - 84.0
**Height** (cm)	169.2 ± 8.5	154.0 - 185.0	170.1 ± 9.5	150.0 - 191.0
**Mass** (kg)	74.5 ± 16.3	47.0 - 104.0	73.3 ± 14.0	47.0 - 104.0
BMI*	25.9 ± 4.5	18.6 - 33.5	25.0 ± 3.3	20.1 - 33.1

*BMI = mass(kg)/height(m)^2^.

For the stroke group, the average time (in months) since stroke was 66.8 months (±SD = 75.3, 6–360). The ratio of involved side (left: right) was 21:8. The Fugl-Meyer Assessment lower limb score mean was 25 (±SD = 5.5) out of a possible 34. Mean maximum strength (1-RM) for the control limb was 70.9 (±SD: 18.1) kg, the uninvolved stroke limb was 60.1 (±SD: 16.6) kg and for the involved stroke limb was 46.4 (±SD: 20.3) kg.

### Peak power

**Figure 2:**
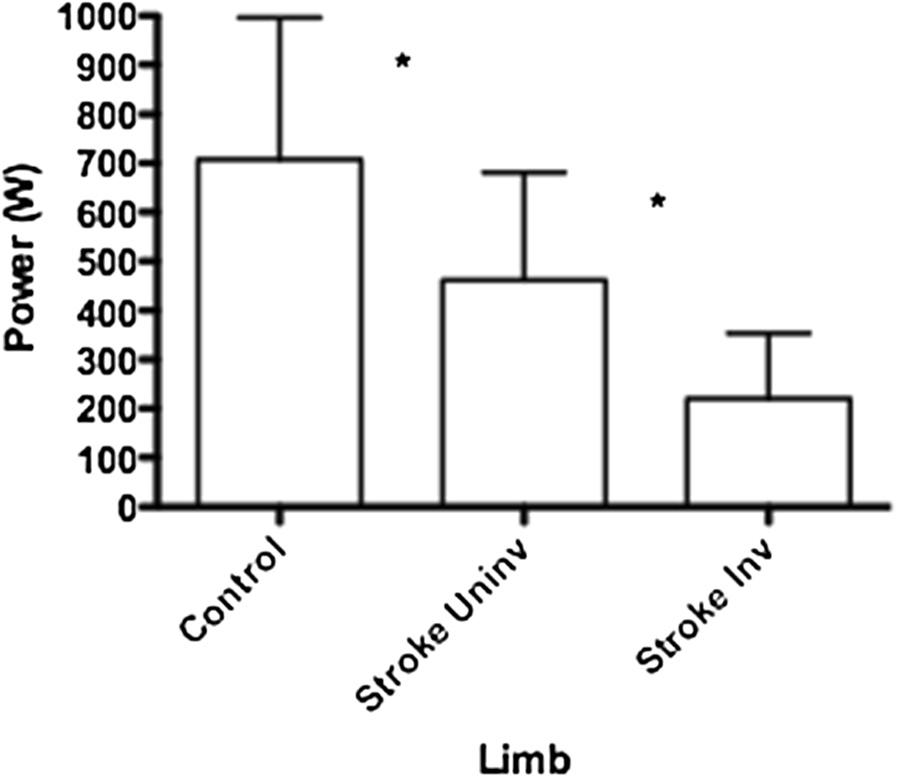
** Peak power (W) and SD across the three limbs irrespective of load shows a significant decrease between all limbs.** (Uninv = uninvolved limb, Inv = involved limb) *p < 0.05.

Figure 2 presents peak power values across limbs irrespective of load. The control group limbs (Mean: 708 ± SD: 289 W) were significantly more powerful (35%) than the uninvolved limbs (Mean: 466 ± SD: 220 W) of the stroke participants. Furthermore, the uninvolved limbs of those with stroke were significantly more powerful (48%) than the involved limbs (Mean: 220 ± SD: 134 W).

**Figure 3:**
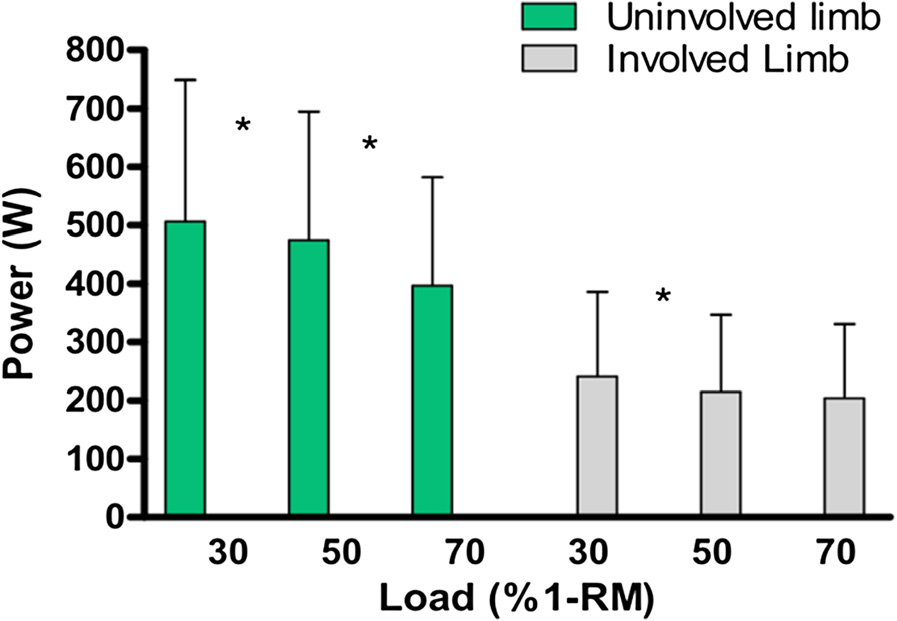
Peak power (W) over 30%, 50%, 70% of 1-RM for the uninvolved and involved limbs of the stroke group showing significant differences across all load levels except between 50 and 70% in the involved limb (*p < 0.05).

Figure 3 shows peak power values across loads and limbs of the stroke group. In both limbs, power declined with increasing load. There was a significant interaction between limbs and load. In the uninvolved limb, peak power was significantly different across all three loads. In the involved limb, there was a significant difference between the 30% of 1-RM and 50% of 1-RM loads but not between 50% of 1-RM and 70% of 1-RM loads.

**Figure 4:**
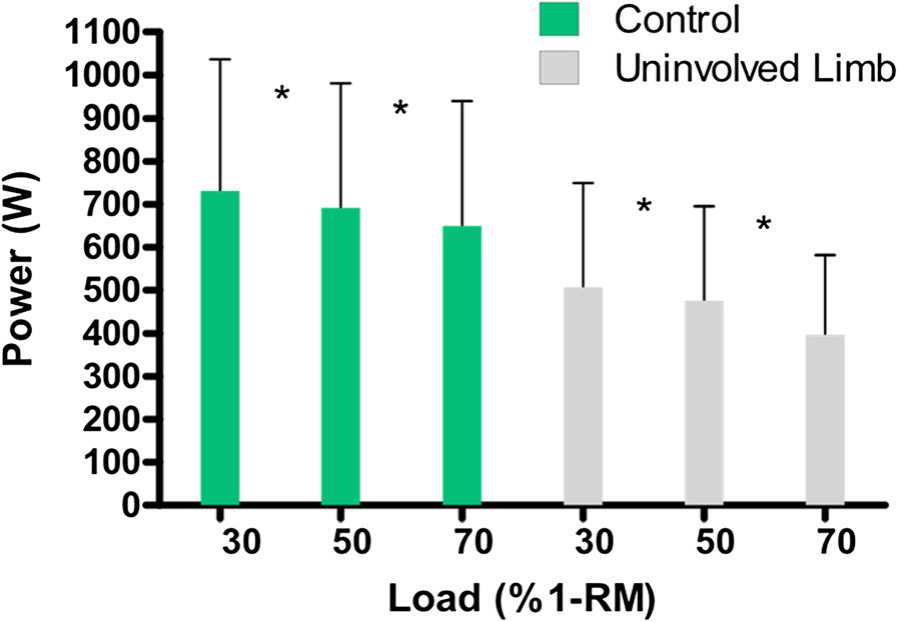
Peak power (W) over 30%, 50%, 70% of 1-RM for the comparison limb of the control group and the uninvolved limb of the stroke group showing significant differences at all loading levels (*p < 0.05).

Figure 4 presents peak power values across loads of the uninvolved limbs of the stroke group and the comparison limb from the control group. In both groups, peak power declined with increasing load and was significantly different across all three loads.

## Discussion

With goals of improving performance of activities and participation levels after stroke, rehabilitation efforts need to consider how improving muscle power may be of benefit [20,21]. In the current study, there were significant differences in peak power across limbs tested regardless of the load at which they pushed*.* The stroke group’s ability to produce peak power in the involved limb was significantly lower than that of the uninvolved limb. This finding has been noted in previous studies that have carried out bilateral lower limb comparison of power in individuals with stroke [20,21,32]. For instance, Dawes et al. [20] noted that the uninvolved limb was 48% more powerful than the involved limb while Saunders et al. [21] showed an 11% difference.

A number of physiological studies provide indirect evidence of potential contributing factors that might explain the deficit in power across legs. Cortical damage, particularly in the motor areas, leads to deficits in voluntary muscle activation [5,33,34] and a subsequent loss of functioning motor units [35]. The remaining motor units demonstrate reduced discharge rates and prolonged contraction times [36-38]. As such, the inability to fully activate existing motor units that are already compromised in recruitment and firing behavior will affect power generation. Furthermore, reductions in both muscle cross-sectional area [39,40] and in Type II muscle fiber numbers [36,41] would further contribute to the power deficits seen. As well, reduced muscle fiber lengths [42] lead to less capacity to generate force at higher muscle shortening velocities and also at greater muscle lengths [43]. As such, it is apparent that a combination of factors has the potential to contribute to the deficit in power observed across limbs in our stroke group.

No other studies have compared the uninvolved limbs of a stroke group to a comparison limb of a control group without stroke. Power differed significantly and was 35% lower in the uninvolved hemiplegic leg, a finding that reflects a pattern also seen with strength loss [5,33].

In addition to the physiological variables mentioned above, differences in power across control and stroke groups may also reflect psychological factors such as self-efficacy and motivation [7]. However, the participants in the current study were self motivated to participate and, hence, might be regarded as a group without a notable loss of self efficacy or motivation. Strong verbal encouragement was also provided consistently to both groups during the performance of all the assessments.

With an established relationship between muscle power and function [12-16,21], rehabilitation efforts need to address the impact of these power deficits on not only the ‘involved’ but also the ‘uninvolved’ limb if there is to be an impact on activity performance. At present, if one followed a traditional model of rehabilitation, then the deficit in power could be corrected to within 10% of the uninvolved side. Such a prescription, however, may not address the uninvolved limb deficits, resulting in continued impairment and activity limitation in comparison to those who have not suffered a stroke. Thus, training of a bilateral nature is suggested in this population in an effort to optimize power levels that would contribute to enhancing function during activities of daily living, work and leisure.

Our results demonstrated that peak power decreased as load increased from 30% of 1-RM to 70% of 1-RM in both stroke and control limbs. This pattern is similar to studies investigating peak power in younger adults performing lower limb extension and jumping exercises [44-47]. It has also been shown that weaker or untrained participants, perhaps more in line with our participants, generally achieve peak power at lower loads [48-50]. However, not all studies agree with such findings. Some authors investigating muscle power in the older adult have reported that power is maximized at higher percentages of 1-RM (65%-75% of 1-RM) [11,13,14,18,24,51]. A notable limitation with these studies is that all these authors applied a power testing protocol that was non-randomized and began at 40% of 1-RM with progressive increments until 90% of 1-RM was reached. Since there was no randomization of load and only one trial at each load was performed, the possibility that a learning or familiarization effect by 70% of 1-RM (eg: 4 attempts) cannot be discounted. As such, the participants would have had the opportunity to practice and were generating their greatest power at the higher loads.

The design of the current study does not allow for the identification of optimal level at which to enhance power through training. However, it does allow one to appreciate where the greatest power deficit across loading levels occurs. Although all levels were affected with considerable deficits, given the limited amount of rehabilitation sessions available to individuals with stroke, the need to prioritize training is paramount. Hence, it would seem most appropriate to target lower loading levels (eg: 30% 1RM) as this was where the greatest deficit occurred (See Figure 3). Intervention trials are needed to determine which training loads are most appropriate for improving specific activities that are of most importance to those with stroke.

An advantage of the equipment used in the current study is that it allowed the participants to project themselves off the foot plate, optimizing velocity, similar to that which occurs in many activities of daily living [44,47,52]. However, the equipment does have some limitations. Firstly, strength and power were assessed using a whole leg press action. As such, the specific contribution of the different joints and muscles cannot be determined. Nevertheless, using a leg press action allowed the assessment of a movement pattern that was more generalizable to a number of everyday activities. Another consideration is that the supine leg press machine required participants to lie supine and thus allowed them to exert maximum effort and produce peak power under safe circumstances. However, the power values obtained may not have been reflective of those required in day to day activities, when participants are upright and having to synergistically maintain posture, coordinate their limbs, and maintain balance, all of which are significant issues in people who have sustained a stroke.

## Conclusions

Community dwelling older adults with and without stroke demonstrated significant differences in muscle power performance across limbs confirming that while one side may be significantly affected after stroke, there are bilateral effects in muscle performance following stroke. Secondly, for both groups, lower limb maximum power was produced at lighter loads. Overall, the results provide information that can help guide clinicians prescribing exercise training programs for those with stroke.

## Competing interests

The authors declare that they have no competing interests.

## Authors' contributions

VS prepared subjects, carried out the experiments, collected and analyzed the data, and drafted the manuscript. PJM designed the experiments, helped analyze the data and helped draft the manuscript. Both authors read, edited, and approved the final manuscript.
